# Facilitating Trust Calibration in Artificial Intelligence–Driven Diagnostic Decision Support Systems for Determining Physicians’ Diagnostic Accuracy: Quasi-Experimental Study

**DOI:** 10.2196/58666

**Published:** 2024-11-27

**Authors:** Tetsu Sakamoto, Yukinori Harada, Taro Shimizu

**Affiliations:** 1 Department of Diagnostic and Generalist Medicine, Dokkyo Medical University, 880 Kitakobayashi, Mibu-cho, Shimotsuga-gun, Tochigi, 321-0293, Japan, 81 282-86-1111, 81 282-86-4775

**Keywords:** trust calibration, artificial intelligence, diagnostic accuracy, diagnostic decision support, decision support, diagnosis, diagnostic, chart, history, reliable, reliability, accurate, accuracy, AI

## Abstract

**Background:**

Diagnostic errors are significant problems in medical care. Despite the usefulness of artificial intelligence (AI)–based diagnostic decision support systems, the overreliance of physicians on AI-generated diagnoses may lead to diagnostic errors.

**Objective:**

We investigated the safe use of AI-based diagnostic decision support systems with trust calibration by adjusting trust levels to match the actual reliability of AI.

**Methods:**

A quasi-experimental study was conducted at Dokkyo Medical University, Japan, with physicians allocated (1:1) to the intervention and control groups. A total of 20 clinical cases were created based on the medical histories recorded by an AI-driven automated medical history–taking system from actual patients who visited a community-based hospital in Japan. The participants reviewed the medical histories of 20 clinical cases generated by an AI-driven automated medical history–taking system with an AI-generated list of 10 differential diagnoses and provided 1 to 3 possible diagnoses. Physicians were asked whether the final diagnosis was in the AI-generated list of 10 differential diagnoses in the intervention group, which served as the trust calibration. We analyzed the diagnostic accuracy of physicians and the correctness of the trust calibration in the intervention group. We also investigated the relationship between the accuracy of the trust calibration and the diagnostic accuracy of physicians, and the physicians’ confidence level regarding the use of AI.

**Results:**

Among the 20 physicians assigned to the intervention (n=10) and control (n=10) groups, the mean age was 30.9 (SD 3.9) years and 31.7 (SD 4.2) years, the proportion of men was 80% and 60%, and the mean postgraduate year was 5.8 (SD 2.9) and 7.2 (SD 4.6), respectively, with no significant differences. The physicians’ diagnostic accuracy was 41.5% in the intervention group and 46% in the control group, with no significant difference (95% CI −0.75 to 2.55; *P*=.27). The overall accuracy of the trust calibration was only 61.5%, and despite correct calibration, the diagnostic accuracy was 54.5%. In the multivariate logistic regression model, the accuracy of the trust calibration was a significant contributor to the diagnostic accuracy of physicians (adjusted odds ratio 5.90, 95% CI 2.93‐12.46; *P*<.001). The mean confidence level for AI was 72.5% in the intervention group and 45% in the control group, with no significant difference.

**Conclusions:**

Trust calibration did not significantly improve physicians’ diagnostic accuracy when considering the differential diagnoses generated by reading medical histories and the possible differential diagnosis lists of an AI-driven automated medical history–taking system. As this was a formative study, the small sample size and suboptimal trust calibration methods may have contributed to the lack of significant differences. This study highlights the need for a larger sample size and the implementation of supportive measures of trust calibration.

## Introduction

Diagnostic errors pose a significant problem for the maintenance of high-quality medical care [1], especially in the outpatient setting. In the United States, approximately 5% of outpatients were likely to encounter diagnostic errors [2]. Recent data from Japan indicate that 3.9% of patients in primary care outpatient clinics have experienced diagnostic errors during the last decade [3]. Thus, innovative approaches to improve diagnostic accuracy and minimize errors should be explored and adopted.

The implementation of artificial intelligence (AI)–driven automated medical history–taking systems with differential diagnosis generators is a promising solution, as these systems provide a list of potential differential diagnoses before the information is collected by physicians, thereby aiding more accurate diagnoses [4]. However, AI-related diagnostic errors have become a problem [5]. Among the multiple factors contributing to the diagnostic errors arising from AI implementation, the insufficient accuracy of AI systems is a prominent issue. For example, the diagnostic accuracy of AI-based differential diagnoses for trauma and musculoskeletal disorders was 73% [6]. Another study revealed that the diagnostic accuracy of AI was 53% in patients who visited an outpatient internal medicine department and required hospitalization within 14 days [7]. These reports indicate that AI alone is insufficient for a definitive diagnosis.

Nonetheless, AI-based diagnostic decision support systems can enhance diagnostic accuracy among physicians and medical students [8], which has implications for clinical practice and medical education. However, concerns exist that inexperienced doctors may overly rely on AI diagnosis, even when the AI-provided diagnosis is incorrect [9]. A recent study indicated that biased AI decreased the diagnostic precision of physicians and that providing explanations for AI reasoning did not improve the diagnostic precision [10]. For the effective and safe implementation of AI-based diagnostic decision support systems in clinical settings, it is imperative to focus on two critical aspects: enhancing the diagnostic accuracy of AI systems and facilitating the development of physicians’ skills to evaluate the certainty levels of AI-generated diagnoses.

Prior research outside the health care domain has demonstrated the usefulness of “trust calibration,” which appropriately adjusts trust levels according to the reliability of an AI system [11]. In a drone simulation study, trust calibration prevented people from excessively trusting AI, leading to performance improvements [11]. However, in the medical field, previous studies examining the effectiveness of diagnostic decision support systems have not investigated the relationship between physicians’ final decisions using AI and their trust in the accuracy of AI-based diagnoses [4,7,9]. Similarly, it is unclear whether assessing the accuracy of AI judgments will improve diagnostic safety when using AI-based diagnostic decision support systems.

Therefore, in this study, we aimed to examine whether physicians’ trust calibration for AI-based diagnostic decision support systems improves their diagnostic accuracy.

## Methods

### Ethical Considerations

This study was conducted in accordance with the Declaration of Helsinki and was approved by the Research Ethics Committees of Dokkyo Medical University (R7112J) and Nagano Chuo Hospital (NCR202209). This study involved human subjects and adhered strictly to ethical research standards. All participants provided written informed consent prior to their involvement in the study. They were fully informed about the study’s procedures, its purpose, the voluntary nature of their participation, and their right to withdraw at any time without consequence. To protect participants’ privacy and confidentiality, all personal data were anonymized, and access to this data was restricted to researchers directly involved in the study. There was no financial compensation for participation. No identifiable images of individual participants appear in the manuscript or supplementary material in Multimedia Appendix 1.

### Study Design

This quasi-experimental study was conducted at the Dokkyo Medical University, Japan between August 9 and September 25, 2023.

### AI-Driven Automated Medical History–Taking System

In this study, we used medical history data recorded by AI Monshin, an AI-driven automated medical history–taking system widely used in more than 1,400 medical facilities in Japan. AI Monshin is a software that converts data entered by the patient on a tablet device into medical terms and summarizes them as medical history to provide the top 10 differential diagnoses. In the waiting room, patients entered their age, sex, and free-form description of their symptoms on a tablet. The AI software then selects approximately 20 questions tailored to the patient, which are presented sequentially on a tablet, and patients respond by choosing their answers from the displayed options. Questions were optimized according to past answers and a list of the most relevant candidate differential diagnoses was generated. Additional details of the AI Monshin have been described previously [4].

### Case Creation

Twenty written clinical cases were created based on the medical histories recorded by an AI-driven automated medical history–taking system from actual patients who visited Nagano Chuo Hospital, a community-based hospital in Japan. The following cases were selected. First, we included patients aged 18 years or older who used the AI-driven automated medical history–taking system at the outpatient department of Nagano Chuo Hospital between May 1, 2019, and April 30, 2022, followed by hospitalization within 30 days. Patients without a confirmed diagnosis, those for whom the AI-driven automated medical history–taking system did not list the differential diagnosis, and those who refused to use their data in this study were excluded. The requirement for informed consent from the patients was waived by the research ethics committee. Based on these criteria, 381 cases were stored for case creation. Second, we extracted data and the final diagnosis from the medical history recorded by the AI-driven automated medical history–taking system. Third, the final diagnosis was coded using the *International Classification of Diseases 11th Revision* [12]. The five most frequent disease categories were digestive, circulatory, and respiratory system diseases; neoplasms; and certain infectious or parasitic diseases. Fourth, two researchers (T Sakamoto and YH) independently determined whether the final diagnosis was included within the AI-generated list of differential diagnoses; any inconsistencies were resolved by discussion. The accuracy of the AI differential diagnosis list was 172/381 (45.1%). Fifth, two researchers (T Sakamoto and YH) independently classified the commonality of the final diagnosis (common or uncommon disease) and the typicality of the clinical presentation (typical or atypical presentation). Any inconsistencies were resolved by discussion: an uncommon disease was defined as a disease affecting less than 1 per 2000 people [13], and judged based on the epidemiological data described in UpToDate [14], DynaMed [15], or other scientific literature. Moreover, a typical or atypical presentation was ascertained by referencing descriptions regarding each disease in UpToDate. We included this variable because atypical presentations have been identified as a risk factor for diagnostic errors [16] and could also be a confounding factor in the results of our study. A total of 381 cases were classified into 4 categories: typical presentation of common disease (n=205, 53.8%), atypical presentation of common disease (n=52, 13.7%), typical presentation of uncommon disease (n=93, 24.4%), and atypical presentation of uncommon disease (n=31, 8.1%). Finally, based on the distribution of the disease category, commonality, and typicality in the patient population, we selected 20 cases. Each AI-generated list of differential diagnoses does not necessarily include the correct final diagnosis. We set an even distribution between the cases in the AI-generated list. This was done to prevent automation bias. Table 1 provides detailed information on the distribution of the 20 cases.

**Table 1.** Selected cases based on the distribution of disease category, commonality, and typicality in the patient population.

Case	Typicality	Commonality	AI’s^a^ answer
Typhus fever due to *Orientia tsutsugamushi*	Typical	Uncommon	False
Acute myocardial infarction	Typical	Common	True
Hepatocellular carcinoma of the liver	Atypical	Uncommon	False
Acute appendicitis	Typical	Common	True
Acute pancreatitis	Typical	Uncommon	False
Pneumonitis due to inhalation of food or vomit	Typical	Common	False
Gastroenteritis due to *Campylobacter*	Typical	Common	True
Herpes zoster	Typical	Common	False
Congestive heart failure	Typical	Common	False
Acute pyelonephritis	Typical	Common	False
Polymyalgia rheumatica	Typical	Uncommon	True
Type 2 diabetes mellitus	Atypical	Common	True
Bacterial pneumonia	Atypical	Common	False
Pneumothorax	Typical	Uncommon	True
Pulmonary hypertension	Typical	Uncommon	False
Malignant neoplasm of the pancreas	Typical	Uncommon	True
Cerebral ischemic stroke	Typical	Common	True
Ischemic colitis	Atypical	Uncommon	True
Malignant neoplasms of the stomach	Typical	Common	False
Fracture of the spine	Typical	Common	True

a AI: artificial intelligence.

### Participants and Procedure

We recruited current and former physicians affiliated with the Department of Diagnostic and Generalist Medicine at Dokkyo Medical University Hospital, Japan. It is the referral department for consultations from within and outside the hospital for difficult-to-diagnose cases, one of its specialized tasks. Physicians who refused to participate were excluded. In this study, physicians were assigned by T Sakamoto to either the intervention or control group (1:1) using a computerized randomization process stratified by postgraduate year (PGY). The participants were not informed about whether they were assigned to the intervention group or the control group. Regardless of the group, each physician was requested to read 20 written clinical cases, arranged randomly, with the AI’s list of 10 differential diagnoses, and then give 1 to 3 possible diagnoses (free text) for each case within 3 minutes. The 3-minute time allocated per case was derived from the assumption that physicians usually take less than 3 minutes to consider differential diagnoses from the AI-driven notes with a list of differential diagnoses in daily clinical practice. It was assumed that if the AI-based system could be used in a hospital outpatient setting and took only 3 minutes, it would be useful in practice. In the intervention group, physicians were presented with the statement “Please consider whether the correct diagnosis is included in AI’s list of differential diagnoses,” in addition to receiving the information and the list of the AI’s 10 differential diagnoses. They were then asked whether they believed that the final diagnosis was included in the AI-generated list of 10 differential diagnoses (Yes or No). This intervention served as the trust calibration in this study. To ensure successful collaboration between users and AI, the users need to adjust their trust level according to the actual reliability of the AI, a process called trust calibration [11]. Trust calibration was designated as “correct” when the physicians’ judgment was correct on whether the final diagnosis was included in the AI’s list of 10 differential diagnoses in the intervention group. Meanwhile, in the control group, there was no mention of “Please consider whether the correct diagnosis is included in AI’s list of differential diagnoses.” They were also not asked whether they believed that the final diagnosis was included in the AI-generated list of 10 differential diagnoses.

After responding to all the cases, physicians in both groups were queried, “What level of diagnostic accuracy would you anticipate for this AI Monshin’s list of differential diagnoses?” They were also instructed to rate their contrast level against the AI on a scale of 0% to 100%, defined as the level of confidence in the AI. Using this confidence level, we examined the accuracy of the physician trust calibration.

### Data Collection and Outcomes

We collected data on the physicians’ age, sex, PGY, answers to 20 clinical cases, and confidence level in AI. The primary outcome was the physicians’ diagnostic accuracy. Each physician’s score was evaluated based on 20 questions, with each question worth 1 point. The physicians’ diagnostic accuracy was determined by whether the final diagnosis matched any diagnosis in the physicians’ list of differential diagnoses. The average scores of the two groups were compared. The secondary outcome measure was the physicians’ correctness of trust calibration. The extent to which physicians trusted AI Monshin’s list of differential diagnoses was assessed after adjusting for confounding factors. Two researchers (T Sakamoto and YH) independently evaluated the primary and secondary outcomes, and inconsistencies were resolved through discussion.

### Sample Size Calculation

As the physicians’ diagnostic accuracy was 57.4% in an experimental study using the same AI-based system [9], we assumed a 55% diagnostic accuracy for physicians in the control group. No previous study has investigated the effect size of trust calibration on the physicians’ diagnostic accuracy. Therefore, we assumed that a 15% increase in physicians’ diagnostic accuracy through trust calibration was clinically significant. With this assumption, we calculated a sample size based on a 2-tailed Student *t* test, *α*=.05, power 0.8, allocation ratio 1:1, and SD 0.1, which resulted in the required sample size (number of participating physicians) of 9 per group (total n=18). Considering dropouts during the study, we determined that 20 physicians were required to participate.

### Statistical Analysis

Continuous variables were presented as medians with interquartile ranges and compared between the two groups using the Student *t* test. Categorical variables were presented as numbers and percentages and were compared using the *χ*^2^ test. The primary outcome, physicians’ diagnostic accuracy, was compared between the two groups using the Student *t* test. The secondary outcome, the correctness of trust calibration in the intervention group, was calculated from the number of cases out of 200 where the physician could correctly distinguish whether the final diagnosis was included in the AI list of the 10 differential diagnoses. Furthermore, we used a multivariate logistic regression model to evaluate the accuracy of trust calibration on the diagnostic accuracy of physicians in the intervention group, adjusted for other factors such as disease commonality, disease typicality, sex, and PGY. The confidence level for AI was compared between the two groups using the Student *t* test. All *P* values in the statistical tests were 2-tailed, and *P* values <.05 were considered statistically significant. All statistical analyses were performed using R version 4.3.2 (The R Foundation for Statistical Computing).

## Results

**Figure 1.**
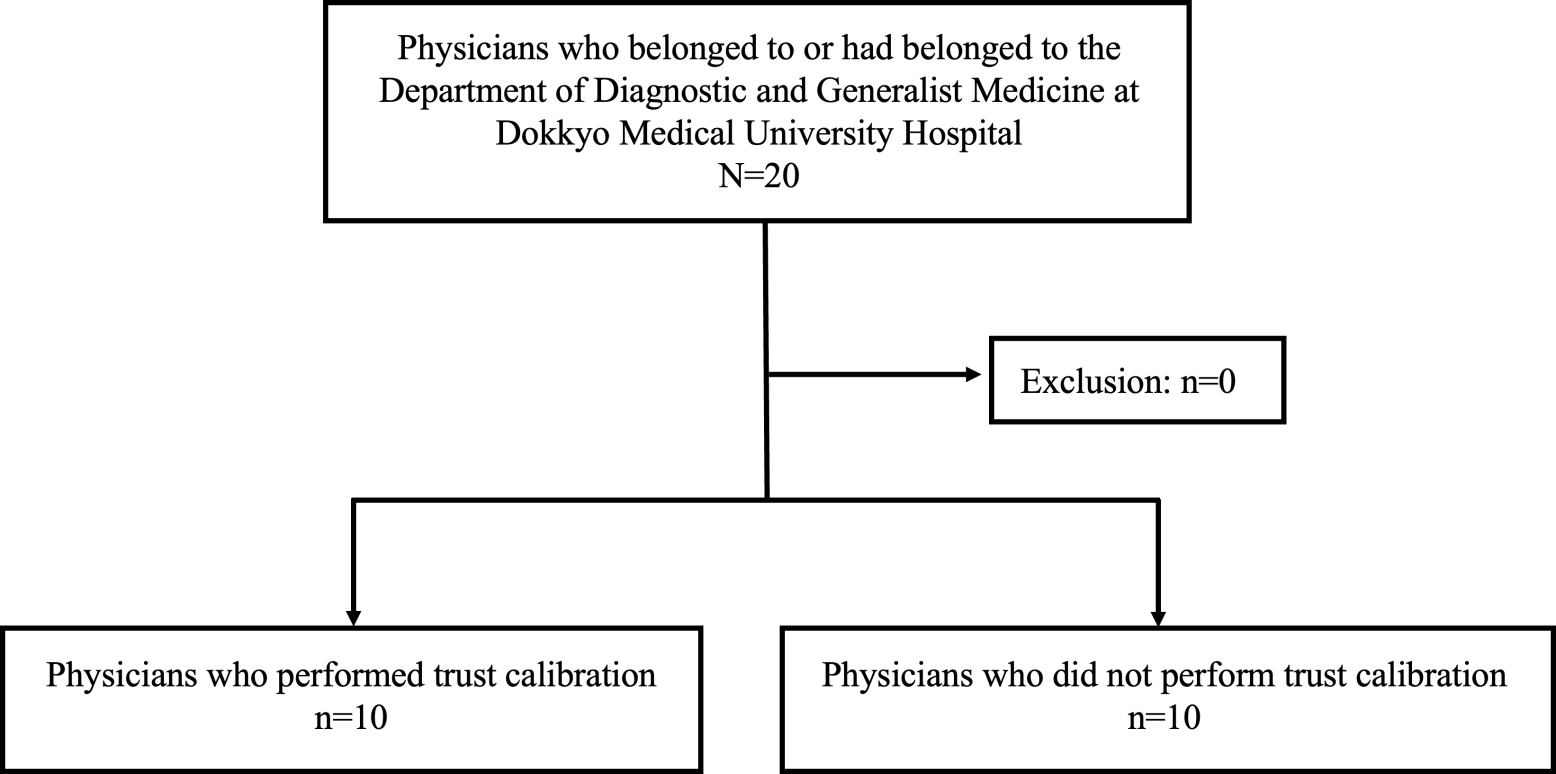
A quasi-experimental study was conducted at Dokkyo Medical University, Japan, with general physicians allocated (1:1) to the intervention and control groups to examine whether physicians’ trust calibration for AI-based diagnostic decision support systems improves their diagnostic accuracy.

Twenty physicians were included and assigned to the intervention (n=10) and control (n=10) groups, and there was no dropouts (Figure 1). The characteristics of the intervention and control groups were as follows: the mean age was 30.9 (SD 3.9) years and 31.7 (SD 4.2) years, the proportion of men was 80% (8/10) and 60% (6/10), and the mean PGY was 5.8 (SD 2.9) and 7.2 (SD 4.6), respectively. There was no significant intergroup difference in the baseline characteristics of the physicians.

### Evaluation Outcomes

The primary outcome, physicians’ diagnostic accuracy, was detected in 8.3 of 20 answers (41.5%) in the intervention group and in 9.2 of 20 answers (46%) in the control group. There was no significant intergroup difference in the physicians’ diagnostic accuracy (95% CI −0.75 to 2.55; *P*=.27).

The secondary outcome, the correctness of trust calibration in the intervention group, was 61.5% (123/200; Table 2). The physicians’ diagnostic accuracy was 54.5% (67/123) in cases where trust calibration was correct, and 20.8% (16/77) in cases where trust calibration was incorrect. The accuracy of trust calibration was a significant contributor to the diagnostic accuracy of physicians (adjusted odds ratio 5.90, 95% CI 2.93‐12.46; *P*<.001) in the multivariate logistic regression model (Table 3).

The confidence level for AI was 72.5% (10%‐80%) in the intervention group and 45% (30%‐80%) in the control group. There was no statistically significant intergroup difference (*P*=.12). The results are shown in Table 4.

**Table 2.** Association between artificial intelligence (AI)’s answer and trust calibration.

Trust calibration	AI’s answer		Sum
	Correct	Incorrect	
Correct	87	36	123
Incorrect	13	64	77

**Table 3.** Multivariate logistic regression analysis regarding the diagnostic accuracy of physicians.

Category	Odds ratio	95% CI	*P* value^a^
Physician variables			
Sex (male)	1.08	0.03‐0.46	.87
Years of postgraduation	1.06	0.93‐1.20	.38
Case variables			
Disease commonality	2.72	1.36‐5.57	<.001
Disease typicality	21.67	6.06‐139.25	<.001
Trust calibration	5.90	2.93‐12.46	<.001

a *P* values from multivariable logistic regression.

**Table 4.** Trust levels for AI in the intervention and control groups.

Trust level (%)	Intervention group, n	Control group, n
0-10	1	0
20-30	0	2
40-50	2	6
60-70	3	1
80-90	4	1

## Discussion

### Principal Results

This study showed that physicians’ diagnostic accuracy did not differ between groups with or without trust calibration when considering the differential diagnoses by reading the medical history and lists of possible differential diagnoses of an AI-driven automated medical history–taking system.

### Comparison With Prior Work

In this study, the intervention with trust calibration for AI was not associated with an increase in the diagnostic accuracy. There are several possible reasons for this observation. First, there is a possibility that the trust calibration method is incorrect. In a previous study using drone simulators, the system issued warnings when people exhibited excessive confidence in the AI as its accuracy decreased [11]. In this study, there was no material or warning to help physicians ascertain whether they were overly trusting, which did not improve the physicians’ diagnostic accuracy. Second, an automation bias may have influenced the results. Recent studies have suggested that excessive reliance on AI-based diagnostic-support tools may have adverse outcomes [17,18]. In this study, physicians in the intervention group estimated the accuracy of the AI to be approximately 30% higher than the actual accuracy. As shown in Table 4, other than one outlier with a 10% confidence level in AI, the intervention group showed a tendency for excessive confidence in AI compared to the control group. The outlier was involved in research on the diagnostic accuracy of generative AI, which may have influenced the group’s level of confidence in AI. This result indicates that physicians’ trust calibration of AI without objective indicators may lead to excessive confidence in the AI, resulting in incorrect diagnostic decisions. There are two possible solutions for overcoming excessive confidence in AI systems that aid diagnostic decisions. One is to show physicians the reasoning process of AI-driven diagnostic decision support systems in advance [17], and the other is to utilize another AI that can indicate reliance on AI-driven diagnostic decision support systems [11,19]. Conversely, trust calibration could make it easier for physicians to trust AI, potentially increasing physician satisfaction in clinical decision-making. More accurate trust calibration could improve diagnostic accuracy, leading to better clinical outcomes for patients and creating overall positive effects.

Clinical diagnostic decision support systems have demonstrated effectiveness in medical education as well. Diagnostic decision support systems based on patient history allows for the generation of more accurate differential diagnoses [20]. This finding suggests that the use of clinical diagnostic decision support systems in medical education may increase further in the future. However, excessive reliance on AI-based diagnostic decision support systems could lead to diagnostic errors. Therefore, in addition to improving the accuracy of AI, as suggested by this study, there is a need to develop the ability to evaluate the reliability of AI. It is essential to implement solutions to overcome the excessive trust in AI mentioned earlier.

On multivariate logistic regression analysis, a correlation was observed between the accuracy of trust calibration and physicians’ diagnostic accuracy. This finding suggests the possibility of improving physicians’ diagnostic accuracy if they are provided with a more precise trust calibration. Additionally, the study results showed that a higher diagnostic accuracy was observed in the common and typical presentations of the cases.

### Limitations

This study had several limitations. First, it is unclear whether trust calibration was absent in the control group. Physicians often employ a dual-process medical decision-making model, which incorporates systems 1 and 2 to arrive at a diagnosis [21]. Therefore, the control group may unconsciously engage in trust calibration as part of this dual-process model; however, further investigation is needed to confirm this hypothesis. Second, the patient data used in this study were collected from only one community hospital in Japan, and the disease frequency, commonality, and typicality may differ in other facilities. Third, the participants were young generalist physicians. Therefore, it is unclear whether these results are applicable to physicians in other specialties or to PGY groups. Additionally, the results may vary because of different cultural backgrounds, varying levels of medical training, and different health care systems. Fourth, the overall diagnostic accuracy of physicians was lower than that observed in previous studies, suggesting that clinical cases are difficult to solve. Fifth, physicians’ trust in AI may vary depending on the type of AI used, which may affect trust calibration. Sixth, because both groups reviewed the differential diagnosis list and supported cognitive reinforcement, there is a possibility of effect modification. Seventh, the sample size was limited. This study was exploratory, involving 20 physicians resolving 20 cases. The small sample size may have contributed to the lack of observed significance. Increasing the sample size could enhance the reliability of the results. To compensate for the lack of power, based on this study, it is estimated that approximately 158 participants would be ideal for the next trial. Eighth, our study is similar to previous trust calibration research in terms of evaluating the accuracy of AI. However, it differs in that participants did not have prior information to determine whether the AI was providing correct answers. In this study, it might have been beneficial to inform participants of the AI’s accuracy beforehand. Therefore, this may not represent accurate trust calibration. In the next trial, the intervention group will be informed of the AI’s diagnostic accuracy before starting the test. The test will be conducted on a computer-based platform, and we will incorporate the trust calibration-specific AI used in the past study [11]. This system will alert participants in the intervention group when they are excessively or insufficiently trusting the AI, allowing for appropriate trust calibration. Ninth, this study suggests that a comprehensive evaluation of whether the AI’s differential diagnosis is correct may be as ineffective as verifying one’s own diagnosis [22]. As previous research found encouraging more specific reflection, such as identifying “where the inconsistencies are,” to be effective [23], applying such methods to the AI’s differential diagnosis list could be a viable approach. Tenth, it is uncertain whether the 3-minute time limit was appropriate. Eleventh, automation bias related to AI may have influenced the results in this study; however, there is currently no clear method to prevent this bias. Twelfth, the potential impacts of trust calibration on other aspects of clinical decision-making, such as patient outcomes and physician satisfaction, were not evaluated. Thirteenth, there are currently no accurate and objective measures to evaluate trust calibration, making this a challenge for future research.

### Conclusions

Trust calibration did not significantly influence the physicians’ diagnostic accuracy in collaboration with the differential diagnosis list generated through AI-assisted medical history, which may, therefore, lack practical application in the real-world clinical setting. Nonetheless, based on past evidence, the introduction of a system that alerts physicians when they place excessive confidence in AI could encourage more precise trust calibration and thereby improve diagnostic accuracy. The significance of this study lies in its clear identification of the limitations of existing trust calibration. The study indicates that applying supportive measures with trust calibration, rather than utilizing only trust calibration, could improve diagnostic accuracy. As this study is formative, further studies incorporating an appropriate sample size and methods for trust calibration are necessary.

## Supplementary material

10.2196/58666 Multimedia Appendix 1 Original ChatGPT transcripts.

## Abbreviations

AI: artificial intelligence
PGY: postgraduate year
