# Supplementary material for: Facilitating Trust Calibration in Artificial Intelligence–Driven Diagnostic Decision Support Systems for Determining Physicians’ Diagnostic Accuracy: Quasi-Experimental Study
Source: JMIR Form Res. 2024 Nov 27;8:e58666. doi: 10.2196/58666 (PMC11612524; doi:10.2196/58666)
Supplement: Multimedia Appendix 1 [file formative-v8-e58666-s001.docx]

Original ChatGPT transcripts

**あなた:**

医学研究論文のrevisionに対するpoint by point responseを考えています。 以下の日本語を英語に直してください。

**ChatGPT:**

もちろんです。日本語の文章を教えていただければ、英語に翻訳します。

**あなた:**

コメントありがとうございます。臨床のセッティングによってAIの精度は異なります。あらゆるセッティングでtrust calibrationが有効であることを評価したいと考え、今回の研究ではあえてAIの精度を伝えませんでした。

**ChatGPT:**

Thank you for your comments. The accuracy of AI varies depending on the clinical setting. We intentionally did not convey the accuracy of AI in this study because we wanted to evaluate the effectiveness of trust calibration in various settings.

**あなた:**

また、過去のドローンシュミレーターを用いたtrust calibrationの研究ではgeneral taskをするAIとtrust calibrationをするAIを同時に駆動させるtwin AIを用いることで、その有効性が示されています。私たちの研究では、既存のAI-based diagnostic support systemsにtrust calibration用のAIを導入することが技術的に困難でした。そこで、今回の研究では医療現場、特に診断分野でのtrust calibrationの有効性を評価するプロトタイプの研究として、より簡便にtrust calibrationを行うことにフォーカスを当てました。つまり、trust calibration専用のAIなしで、AIの診断精度を評価できるか試みたのです。今回の研究結果をもとに、AI-based diagnostic support systemsにtrust calibration専用のAIを導入し、類似した研究を行いたいと考えています。

**ChatGPT:**

Additionally, previous studies on trust calibration using drone simulators have demonstrated the effectiveness of employing twin AIs, where one AI performs general tasks and the other AI conducts trust calibration simultaneously. In our study, it was technically challenging to integrate an AI dedicated to trust calibration into existing AI-based diagnostic support systems. Therefore, this study focused on a prototype evaluation of the effectiveness of trust calibration in clinical settings, particularly in diagnostics, by employing a more straightforward approach to trust calibration. In other words, we attempted to assess the diagnostic accuracy of AI without a dedicated trust calibration AI. Based on the results of this study, we plan to integrate a dedicated trust calibration AI into AI-based diagnostic support systems and conduct similar research.

**あなた:**

コメントありがとうございます。日常診療ではAI-based diagnostic support systems を使用した場合でも、その後に医師が追加で病歴を聴取すること、検査等を施行することで診断精度を上げます。今回はAIによる問診のみであり、診断精度が低くなったと考えます。またatypicalなプレゼンテーションは診断エラーにつながることが知られています(1)。今回の研究でもatypical presentationを含んだvignetteが含まれていたことから、診断エラーに繋がった可能性があります。

**ChatGPT:**

Thank you for your comments. In routine clinical practice, even when AI-based diagnostic support systems are used, physicians often improve diagnostic accuracy by additionally taking medical histories and performing examinations. In this study, since only AI-based medical interviews were conducted, we believe this led to lower diagnostic accuracy. Additionally, it is known that atypical presentations can lead to diagnostic errors (1). In our study, the vignettes included atypical presentations, which may have contributed to diagnostic errors.

**あなた:**

コメントありがとうございます。指摘いただいた通り、サンプルサイズが少なかった可能性があります。リミテーションに記載しました。 リミテーションの最後のパラグラフを確認ください。

**ChatGPT:**

Thank you for your comments. As you pointed out, the sample size might have been too small. We have noted this in the limitations section. Please review the last paragraph of the limitations.

Is there anything specific you need me to adjust or add in the last paragraph of the limitations?

**あなた:**

コメントありがとうございます。メソッドのセクションを修正しました。 Study Design and ParticipantsのパラグラフをAI-driven Automated Medical History-systemおよびCase Creationのパラグラフの後に記載しました。

**ChatGPT:**

Thank you for your comments. I have revised the methods section. The paragraph on Study Design and Participants has been placed after the paragraphs on the AI-driven Automated Medical History System and Case Creation.

Would you like me to translate this response into English, or is there anything specific you would like me to modify or add?

**あなた:**

コメントありがとうございます。 過去の研究ではtrust calibrationを行う際に、general taskを行うAIと、trust calibration専用のAIを同時に駆動するtwin AIモデルを使用していました。今回の研究では、既存のAI-based diagnostic support systemsにtrust calibration用のAIを導入することが技術的に困難でした。そこで、まずは医療現場で、より簡便にtrust calibrationを行うことにフォーカスを当てたプロトタイプの研究として行いました。AIの診断を信頼して良いかどうか判断する材料がなかったため、結果的に、自己キャリブレーションになり、診断精度に有意差が出なかった可能性があります。今回の研究結果をもとに、AI-based diagnostic support systemsにtrust calibration専用のAIを導入し、次の研究に繋げたいと考えています。

**ChatGPT:**

Thank you for your comments. In previous studies on trust calibration, a twin AI model was used, where one AI performs general tasks and another AI is dedicated to trust calibration, operating simultaneously. In this study, it was technically challenging to integrate a dedicated trust calibration AI into existing AI-based diagnostic support systems. Therefore, we conducted a prototype study focusing on a simpler approach to trust calibration in clinical settings. Due to the lack of criteria to judge whether the AI's diagnosis could be trusted, it resulted in self-calibration, which may have led to no significant difference in diagnostic accuracy. Based on the results of this study, we plan to integrate a dedicated trust calibration AI into AI-based diagnostic support systems and conduct further research.

**あなた:**

コメントありがとうございます。今回の研究は医学教育を目的とした研究ではないため、指摘いただいたディスカッションについては割愛させていただきます。

**ChatGPT:**

Thank you for your comments. Since this study is not aimed at medical education, we will omit the discussion points you mentioned.

**あなた:**

コメントありがとうございます。この論文はEditageで精密な英文校正を受けました。

**ChatGPT:**

Thank you for your comments. This paper has undergone precise English proofreading by Editage.

**あなた:**

コメントありがとうございます。指摘いただいた点をリミテーションに追記しました。 リミテーションの中段です。

**ChatGPT:**

Thank you for your comments. We have added the points you mentioned to the limitations section. They are included in the middle part of the limitations.

Is there anything specific you would like me to modify or add?

**あなた:**

コメントありがとうございます。指摘いただいた通りです。コントロール群における潜在的なtrust calibrationの可能性について、リミテーションに記載しています。 リミテーションのセクションの最初に記載。

**ChatGPT:**

Thank you for your comments. As you pointed out, we have noted the potential for implicit trust calibration in the control group. This is mentioned at the beginning of the limitations section.

Would you like any further adjustments or additions to this response?

**あなた:**

既存のAI-based diagnostic support systemsに過去のドローンシュミレーターの研究で使用されたtrust calibration専用のAIの導入や、eye-trackingやphysiological responsesに注目した評価方法により、より正確なtrust calibrationに繋がる可能性がある。

**ChatGPT:**

The integration of a dedicated trust calibration AI, as used in previous drone simulator studies, into existing AI-based diagnostic support systems, along with evaluation methods focusing on eye-tracking and physiological responses, could lead to more accurate trust calibration.

**あなた:**

私たちはあなたのコメントに賛成です。 ディスカッションのセクションの、Comparison with Prior Workのパラグラフ中盤に追記をしました。

**ChatGPT:**

We agree with your comments. We have added this point to the middle of the paragraph in the Comparison with Prior Work section of the discussion.

Would you like any further adjustments or additions to this response?

**あなた:**

一方で、逆説的に言えば、信頼校正を行うことで、医師がAIを信頼しやすくなり、clinical decision-makingにおいて、医師の満足度が上がる可能性がある。より正確な信頼校正を行えば、診断精度も向上し、患者の臨床的な予後の改善にもつながり、全体にとってプラスの作用が生まれる可能性がある。

**ChatGPT:**

Conversely, trust calibration could make it easier for physicians to trust AI, potentially increasing physician satisfaction in clinical decision-making. More accurate trust calibration could improve diagnostic accuracy, leading to better clinical outcomes for patients and creating overall positive effects.

Is there anything specific you would like me to modify or add to this response?

**あなた:**

私たちはあなたのコメントに賛成です。 ディスカッションのセクションの、Comparison with Prior Workのパラグラフ後半に追記をしました。

**ChatGPT:**

We agree with your comments. We have added this point to the latter part of the paragraph in the Comparison with Prior Work section of the discussion.

Would you like any further adjustments or additions to this response?

**あなた:**

これは自動化バイアスを防ぐためでした。

**ChatGPT:**

This was done to prevent automation bias.

**あなた:**

コメントありがとうございます。メソッドではAIの鑑別疾患があっているケースを10、間違っているケースを10用意することで、自動化バイアスの軽減を意識しました。 自動化バイアスを防ぐための戦略については、ディスカッションのComparison with Prior Workのパラグラフの後半に記載してある通りです。 メソッドのCase Creationのパラグラフの終盤に追記をしました。

**ChatGPT:**

Thank you for your comments. In the methods section, we prepared 10 cases where the AI's differential diagnosis was correct and 10 cases where it was incorrect to mitigate automation bias. As mentioned in the latter part of the Comparison with Prior Work paragraph in the discussion, strategies to prevent automation bias are discussed. This point has been added to the end of the Case Creation paragraph in the methods section.

**あなた:**

コメントありがとうございます。日本の市中病院の初診外来には、施設によりますが1日20人程度の患者が来ることが多いです。臨床現場でAI Monshinを使用する場合、AI Monshinの提示する病歴と鑑別診断を確認し、その後、医師が新たに診察を行い、鑑別疾患を考える、という診療の流れになります。患者さん1人にかける時間を考慮すると、医師が自分で行う問診の後に鑑別疾患を考える時間を取るため、AI問診の提示する病歴や鑑別診断は3分程度で確認し、診断サポートになればより実用的であると考え、3分以内、という設定を行いました。

**ChatGPT:**

Thank you for your comments. In typical Japanese community hospitals, approximately 20 patients visit the outpatient clinic daily, depending on the facility. When using AI Monshin in clinical settings, the process involves reviewing the medical history and differential diagnoses presented by AI Monshin, followed by the physician conducting a new examination and considering differential diagnoses. Considering the time spent per patient, it is practical for the physician to spend about three minutes reviewing the medical history and differential diagnoses provided by AI Monshin before formulating their own differential diagnoses. Therefore, we set the time limit to three minutes, aiming to make the AI Monshin more practical as a diagnostic support tool.

**あなた:**

医師が患者さんに問診を行った後、鑑別疾患を考える時間を取るため、患者さん1人にかける時間を考慮すると、信頼校正を含めてAI問診の提示する病歴や鑑別診断は3分程度で確認し、それが診断のサポートになればより実用的であると考え、3分以内、という設定を行いました。

**ChatGPT:**

Considering the time spent per patient and allowing physicians time to formulate differential diagnoses after conducting their own patient interviews, we determined that reviewing the medical history and differential diagnoses provided by the AI, including trust calibration, should take approximately three minutes. We believe that if this process supports diagnosis within this time frame, it would be more practical. Therefore, we set the time limit to three minutes.

**あなた:**

コメントありがとうございます。日本の市中病院の初診外来には、施設によりますが1日20人程度の患者が来ることが多いです。臨床現場でAI Monshinを使用する場合、AI Monshinの提示する病歴と鑑別診断を確認し、その後、医師が新たに診察を行い、鑑別疾患を考える、という診療の流れになります。

**ChatGPT:**

Thank you for your comments. In typical Japanese community hospitals, about 20 patients visit the outpatient clinic daily, depending on the facility. When using AI Monshin in clinical settings, the workflow involves reviewing the medical history and differential diagnoses presented by AI Monshin, followed by the physician conducting a new examination and considering differential diagnoses.

**あなた:**

considering differential diagnosesを言い換えてほしい

**ChatGPT:**

Certainly! Here's a rephrased version:

Thank you for your comments. In typical Japanese community hospitals, about 20 patients visit the outpatient clinic daily, depending on the facility. When using AI Monshin in clinical settings, the workflow involves reviewing the medical history and differential diagnoses presented by AI Monshin, followed by the physician conducting a new examination and developing differential diagnoses.

**あなた:**

過去の信頼校正の研究とは、AIの精度を検証するという点では類似しているが、AIが正しい答えを提示しているか判断する材料がない点は異なる。今回の研究では、参加者に、事前にAIの精度を伝えるべきであった。このため、正確な信頼校正とは言えないかもしれない、。

**ChatGPT:**

Our study is similar to previous trust calibration research in terms of evaluating the accuracy of AI. However, it differs in that participants did not have prior information to determine whether the AI was providing correct answers. In this study, it might have been beneficial to inform participants of the AI's accuracy beforehand. Therefore, this may not represent accurate trust calibration.

**あなた:**

参照した岡村らの研究コンセプトとの類似点は、人がAIの診断精度を検証している点です。一方で、今回の研究ではAIが正しい答えを提示しているか判断する指標がなかったため、それは異なる点です。指摘いただいた通り、今回使用したAIの診断精度が50%であることを事前に参加者に伝えておくべきでした。リミテーションに以下のように記載をしました。

**ChatGPT:**

The similarity with the concept of the study by Okamura et al. lies in the fact that humans are evaluating the diagnostic accuracy of AI. However, our study differs in that there were no indicators for determining whether the AI was providing correct answers. As you pointed out, we should have informed participants in advance that the diagnostic accuracy of the AI used in this study was 50%. This point has been noted in the limitations section as follows.

**あなた:**

今回の研究で使用したAIの診断精度は約50%です。過去の研究で同じ診断精度のAIを利用した際に、医師の診断精度は56%でした。同様に、別の研究で、医師の診察前に意思決定サポートシステムを利用した場合の診断精度は58%でした。診察前のAI-based diagnostic support systemsの使用では高い診断精度にはつながらないことがわかっています。

**ChatGPT:**

The diagnostic accuracy of the AI used in this study is approximately 50%. In previous research, when an AI with the same diagnostic accuracy was used, the diagnostic accuracy of physicians was 56%. Similarly, in another study, the diagnostic accuracy was 58% when a decision support system was used before the physician's examination. These findings indicate that the use of AI-based diagnostic support systems prior to the examination does not lead to high diagnostic accuracy.

**あなた:**

今回の研究は探索的研究として行ったため、指摘いただいた通りサンプル不足の可能性は大いにあります。以下のようにリミテーションに記載しました。

**ChatGPT:**

As this study was conducted as an exploratory research, there is indeed a significant possibility of sample size insufficiency, as you pointed out. We have noted this in the limitations section as follows.

**あなた:**

第7に、サンプル数が少なかったことである。今回は20人の医師に20のケースを解決してもらうという探索的研究であった。サンプル数が少ないために有意差が観察されなかった可能性がある。サンプル数を増やして評価することで、結果の信頼性が高まる可能性がある。

**ChatGPT:**

Seventh, the sample size was limited. This study was exploratory, involving 20 physicians resolving 20 cases. The small sample size may have contributed to the lack of observed significance. Increasing the sample size could enhance the reliability of the results.

**あなた:**

問題の正答率

**ChatGPT:**

Seventh, the sample size was limited. This study was exploratory, involving 20 physicians resolving 20 cases. The small sample size may have contributed to the lack of observed significance in the accuracy of the answers. Increasing the sample size could enhance the reliability of the results.

**あなた:**

主要評価項目は、医師の診断精度、つまり問題の正答率である。

**ChatGPT:**

Seventh, the sample size was limited. This study was exploratory, involving 20 physicians resolving 20 cases. The primary outcome measure was the diagnostic accuracy of the physicians, i.e., the correctness of their answers. The small sample size may have contributed to the lack of observed significance. Increasing the sample size could enhance the reliability of the results.

**あなた:**

以下の文章を英語に訳してください 主要評価項目は、医師の診断精度＝問題の正答率としました。

**ChatGPT:**

The primary outcome measure was the diagnostic accuracy of the physicians, defined as the correctness of their answers.

**あなた:**

コメントありがとうございます。私の文章表現の仕方が悪く、誤って伝わってしまったと思います。大変申し訳ありません。1人の医師が解く20問は、全て異なる問題であるため、repeated measurement ANOVAではありません。プライマリアウトカムの設定とサンプルサイズ計算を以下のように変更しました。プライマリアウトカムは医師の診断精度です。問題20問を1問1点として、1人の医師が何点獲得できたかを評価し、インターベンション群とコントロール群での平均得点を比較することとしました。医師の正答は、最終診断が医師の鑑別診断リストにある診断のいずれかと一致しているかで判定しました。 サンプルサイズは、過去の研究をもとにコントロール群の正答率を55%、インターベンション群の正答率を70%として、SDを0.1に設定し、Sutdent t検定を用いて必要な参加者数を計算しました。 本文の修正内容は以下です。

**ChatGPT:**

Thank you for your comments. I apologize for the misunderstanding caused by my previous explanation. Since each of the 20 questions solved by one physician is different, repeated measures ANOVA was not applicable. We have revised the primary outcome setting and sample size calculation as follows:

The primary outcome measure is the diagnostic accuracy of the physicians. Each of the 20 questions is scored on a scale of 1 point per question, and we evaluated how many points each physician scored. We compared the mean scores between the intervention group and the control group. A physician's correct answer was determined by whether the final diagnosis matched any of the diagnoses listed in the physician's differential diagnosis list.

The sample size calculation was based on previous studies, assuming a correct answer rate of 55% in the control group and 70% in the intervention group, with an SD of 0.1. We used the Student's t-test to calculate the required number of participants.

The revised content of the main text is as follows:

Would you like me to translate the revised text for inclusion in the main document?

**あなた:**

おっしゃる通りです。 今回の研究では、誰かの提案(AIの鑑別診断リスト)を見て正しいかを判断する包括的検証ですが、自身の診断の検証と同様に効果がないことを示唆していると思います。指摘いただいた点について以下のようにリミテーションに記載をしました。

**ChatGPT:**

As you mentioned, this study involved a comprehensive evaluation of determining the accuracy of suggestions (AI's differential diagnosis list), similar to verifying one's own diagnosis, which suggests that it may not be effective. We have noted the points you raised in the limitations section as follows.

**あなた:**

単に「AIの鑑別診断が正しいかどうか」という包括的検証は自身の診断の検証と同様に効果はない可能性があることを今回の研究は示している。「矛盾がある点はどこか」のようにより具体的な振り返りを促すと効果が出たという先行研究を踏まえると、AIの鑑別診断リストについてもそのような手法を用いることも一案である。

**ChatGPT:**

This study suggests that a comprehensive evaluation of whether the AI's differential diagnosis is correct may be as ineffective as verifying one's own diagnosis. Based on previous research that found encouraging more specific reflection, such as identifying "where the inconsistencies are," to be effective, applying such methods to the AI's differential diagnosis list could be a viable approach.

**あなた:**

用語の区別ができていませんでした、申し訳ありません。以下のように修正しました。

**ChatGPT:**

No problem at all. Here is the revised version:

This study suggests that simply conducting a comprehensive evaluation of whether the AI's differential diagnosis is correct may be as ineffective as verifying one's own diagnosis. Based on previous research that showed encouraging more specific reflection, such as identifying "where the inconsistencies are," to be effective, applying such methods to the AI's differential diagnosis list could be a viable approach.

**あなた:**

以下の文章を英語にしてください。 用語の区別ができていませんでした、申し訳ありません。以下のように修正しました。

**ChatGPT:**

I apologize for not distinguishing between the terms correctly. I have revised it as follows.

**あなた:**

医学教育に関連した内容について、イントロダクションの微修正と、ディスカッションの追加を行いました。

**ChatGPT:**

I have made minor revisions to the introduction and added content to the discussion regarding topics related to medical education.

**あなた:**

コメントありがとうございます。今回使用したAI-based diagnostic support systemsは、プレコンサルテーションとして実装されています。過去の研究では時間をかけるほど診断精度が上がるわけではないことが証明されています（Norman G, et al. Academic Medicine. 2014;89(2):277-84）。そのため、プレコンサルテーションであれば3分間は妥当だと感じますが、実際にそれを証明している研究はないことから、真に妥当かはわからないため、リミテーションに以下のように記載しました。

**ChatGPT:**

Thank you for your comments. The AI-based diagnostic support systems we used in this study are implemented as pre-consultation tools. Previous research has shown that spending more time does not necessarily increase diagnostic accuracy (Norman G, et al. Academic Medicine. 2014;89(2):277-84). Therefore, we believe that three minutes is reasonable for pre-consultation use. However, since there are no studies proving this, it is uncertain whether it is truly appropriate. We have noted this in the limitations section as follows.

**あなた:**

3分という時間制限が適切であったかはわからない。

**ChatGPT:**

It is uncertain whether the three-minute time limit was appropriate.

**あなた:**

第10に、

**ChatGPT:**

Tenth, it is uncertain whether the three-minute time limit was appropriate.

**あなた:**

コメントありがとうございます。AIに関連したautomation biasを防ぐ方法については、今の時点では明確な方法はありません。以下のようにリミテーションに記載しました。

**ChatGPT:**

Thank you for your comments. At this point, there is no clear method to prevent automation bias related to AI. We have noted this in the limitations section as follows.

**あなた:**

この研究でもAIに関連したautomation biasが影響した可能性はあるが、このバイアスを防ぐための明確な方法が現時点ではない。

**ChatGPT:**

In this study, automation bias related to AI may have influenced the results; however, there is currently no clear method to prevent this bias.

**あなた:**

第11に、

**ChatGPT:**

ChatGPT

Eleventh, automation bias related to AI may have influenced the results in this study; however, there is currently no clear method to prevent this bias.

**あなた:**

信頼キャリブレーションが医師の満足度にも影響を与える可能性はありますが今回は評価できていませんでした。以下のようにリミテーションに記載しました。

**ChatGPT:**

Trust calibration may also impact physician satisfaction, but we were unable to evaluate this in the current study. We have noted this in the limitations section as follows.

**あなた:**

今後の研究では評価したいと思います。

**ChatGPT:**

We plan to evaluate this in future research.

**あなた:**

信頼キャリブレーションが医師の満足度にも影響を与える可能性はありますが今回は評価できていませんでした。今後の研究で評価したいと思います。以下のようにリミテーションに記載しました。

**ChatGPT:**

Trust calibration may also impact physician satisfaction, but we were unable to evaluate this in the current study. We plan to evaluate this in future research. We have noted this in the limitations section as follows.

**あなた:**

信頼キャリブレーションが臨床意思決定に与える他の潜在的な影響（例えば、患者の結果や医師の満足度）については評価できていない。

**ChatGPT:**

The potential impacts of trust calibration on other aspects of clinical decision-making, such as patient outcomes and physician satisfaction, were not evaluated.

**あなた:**

第12に、

**ChatGPT:**

Twelfth, the potential impacts of trust calibration on other aspects of clinical decision-making, such as patient outcomes and physician satisfaction, were not evaluated.

**あなた:**

コメントありがとうございます。現時点ではトラストキャリブレーションを正確かつ客観的に評価する指標がありません。リミテーションに記載しました。

**ChatGPT:**

Thank you for your comments. Currently, there are no accurate and objective measures to evaluate trust calibration. We have noted this in the limitations section.

**あなた:**

第13に、現時点ではトラストキャリブレーションを正確かつ客観的に評価する指標がないため、今後の課題である。

**ChatGPT:**

Thirteenth, there are currently no accurate and objective measures to evaluate trust calibration, making this a challenge for future research.

**あなた:**

本原稿をJMIR Formative Researchにトランスファーしてもらいました。

**ChatGPT:**

We have transferred this manuscript to *JMIR Formative Research*.

**あなた:**

Reviewer Aのコメントに対応しました。

**ChatGPT:**

I have addressed the comments from Reviewer A.

**あなた:**

今回の研究はformative research　であり、より洗練して次のトライアルをする。その際のサンプルサイズやインターベンションとコントロールグループの対応について、リミテーションセクションに以下のように記載した。

**ChatGPT:**

This study is formative research, and we plan to refine it for the next trial. We have noted the sample size and the handling of the intervention and control groups for the next trial in the limitations section as follows.

**あなた:**

このことから次回はより多くの参加者が必要である。

**ChatGPT:**

Therefore, a larger number of participants will be needed in the next trial.

**あなた:**

仮定したエフェクトサイズ

**ChatGPT:**

the assumed effect size

**あなた:**

日本語にして RESPONSE: The similarity with the concept of the study by Okamura et al. lies in the fact that humans are evaluating the diagnostic accuracy of AI. However, our study differs in that there were no indicators for determining whether the AI was providing correct answers. As you pointed out, we should have informed participants in advance that the diagnostic accuracy of the AI used in this study was about 50%. This point has been noted in the limitations section as follows; Ehghth, our study is similar to previous trust calibration research in terms of evaluating the accuracy of AI. However, it differs in that participants did not have prior information to determine whether the AI was providing correct answers. In this study, it might have been beneficial to inform participants of the AI's accuracy beforehand. Therefore, this may not represent accurate trust calibration.

**ChatGPT:**

以下のように日本語に翻訳します：

RESPONSE: 岡村らの研究コンセプトとの類似点は、人間がAIの診断精度を評価している点にあります。しかし、今回の研究は、AIが正しい答えを提示しているかどうかを判断する指標がなかった点で異なります。ご指摘の通り、今回使用したAIの診断精度が約50％であることを事前に参加者に伝えるべきでした。この点については、以下のようにリミテーションに記載しました。

第8に、今回の研究はAIの精度を評価する点では過去の信頼校正の研究と類似していますが、AIが正しい答えを提供しているかを判断するための事前情報が参加者に提供されていなかった点で異なります。この研究では、参加者に事前にAIの精度を伝えることが有益だったかもしれません。したがって、この研究は正確な信頼校正を表していない可能性があります。
